# Predictors of the Public’s Aversion to Patients Infected with COVID-19 in China: The Mediating Role of Negative Physiology

**DOI:** 10.3390/healthcare10101813

**Published:** 2022-09-20

**Authors:** Ke Zhang, Boya Han, Ran Meng, Jiayi Hou, Long Chen

**Affiliations:** 1 School of Communication, Soochow University, Suzhou 215123, China; 2 Department of Psychology, Renmin University of China, Beijing 100872, China

**Keywords:** social media usage, risk communication, prevention measures, stigma, disgust, avoidance

## Abstract

COVID-19 has had a lasting impact on the public’s mental health. Understanding the mechanism of the formation of the public’s aversion to COVID-19-infected people can not only help eliminate the irrational stigma, rejection, and aversion of the public but also promote the creation of a harmonious and healthy social atmosphere. Based on stimulus–organism–response theory, this study explored the relationships between environmental stimuli, public negative physiology, and aversion responses. A cross-sectional, online-based survey study was conducted in April 2022. A total of 1863 effective questionnaires from respondents of various ages, genders, incomes, and education levels were acquired. Structural equation modeling was used to test the proposed model. The environmental stimuli including the use of social media and the perception of risk communication aggravated the negative physiology of the public, while the public’s perception of prevention measures reduced the public’s negative physiology during the epidemic. The negative physiology of the public increases the public’s aversion responses, including disgust, stigma, and avoidance, toward patients infected with COVID-19. The negative physiology of the public plays a mediating role in the relationship between the environmental stimuli and the public’s aversion to patients infected with COVID-19. The emergence of excessive information in social media and strict prevention measures in daily life, as well as the dissemination of a large amount of risk information in pseudo-environments and realistic environments, have all exerted an impact on public sentiment and cognition. In the case of the prolonged spread of the epidemic, the accumulation of negative physiology, such as anxiety, panic, and depression, is more likely to lead to the public’s aversion to people with COVID-19.

## 1. Introduction

The COVID-19 cases worldwide have exceeded 600 million, and the death toll has exceeded 6.51 million as of September 2022 [1]. The epidemic has been ongoing in most parts of China for more than two years, and the long-term and strict prevention measures have effectively controlled the spread of the epidemic [2]. However, the epidemic has effected in the public negative physiological states such as stress, anxiety, depression, and fear [3]. At an early stage of COVID-19, Wang et al. found that more than 53% of citizens in China felt that the epidemic had had a moderate or even severe negative psychological impact on them [4].

Negative emotional responses such as fear and anger may be related to behavioral tendencies of the public towards the persons infected with COVID-19 [5,6]. For example, Faulkner et al. pointed out that individuals prefer to interact with internal group members and reject contact with external group members to reduce the risk of infection [7]. During the 2014 Ebola outbreak, the countries with the epidemic were stigmatized and rejected by the world, bringing people in these countries anxiety and depression, psychiatric disorders, and post-traumatic stress reactions [8]. Researchers also identified levels and correlates of stigma toward individuals and households who tested positive for COVID-19 in Bangladesh [9]. 

The stimulus–organism–response (SOR) model was first proposed by Mehrabian and Russell to explain the effects of external environmental stimuli (S) on individuals’ emotions and cognition (O) and to predict individuals’ responses (R) based on changes in emotions and cognition [10]. The SOR model shows a more dynamic process from external environmental stimuli to the individual’s organismic response to the formation of behavioral responses, and it better reflects the changes in the individual’s psychological and behavioral intentions in the situation.

The SOR model has mostly been applied to exploring the influence of environmental factors on the psychological and physiological responses of individuals. For example, Wang et al. utilized the SOR theory to explore the relationship between the stimulus of a major public health emergency and the emergency psychology of the masses, as well as the emergency actions of the masses, in the context of the COVID-19 pandemic [11]. Pan and Lu investigated the factors influencing user engagement behavior in online health communities and explored the effect of social support on user engagement behavior based on the mediating role of body emotions based on the SOR model [12]. Wang et al. discussed the reality of primary health services to analyze how residents’ health management status and the environmental factors stimulated by an image of the medical unit’s grooming—through the organismic response stimulation of the residents’ basic information status—affected the residents’ willingness to make the first visit to primary care [13]. 

In the SOR theoretical model, S (stimulus) denotes the external environmental stimuli. The public is exposed to two types of information related to the COVID-19 pandemic: online information in the mimetic environment and prevention information that can be perceived by the public in the real environment. Three variables, social media usage, risk communication, and prevention measures, were used to measure the intensity of external stimuli in this study. Social media usage tends to be the information stimulus in the mimetic environment, prevention measures tend to be the information stimulus in the real environment, and risk communication includes the information received by individuals in the virtual and real environments. O (organism) represents the organism, i.e., the cognitive and psychological state of the individual, and the variable negative physiology is used as the organism element in this study. R (response) represents the willingness and reaction formed based on the cognitive and psychological levels. The willingness of an individual is a necessary precondition for the generation of a specific behavior, and it reflects the individual’s behavior to a certain extent. In previous studies on SOR theory, willingness to accept, willingness to pay, and willingness to travel for recreation were considered response factors [14]. The nature of the aversion to novel coronavirus-infected persons studied in this paper belongs to the category of public will, which is the antecedent response to the actual behavioral actions of the public toward novel coronavirus-infected persons in real life. Therefore, this study includes the public’s aversion to novel coronavirus infections in the response category.

Several studies paid attention to mass psychology in the context of COVID-19, investigating the psychological distress of people including their fear, loneliness, conflict, and anger in the epidemic situation [15,16,17,18]. These studies provided suggestions for reducing mental disorders, including strengthening family communication, seeking social support, dispelling rumors related to the epidemic, and developing psychosocial services [19,20,21]. 

To the best of the authors’ knowledge, no study has been performed to investigate the public’s attitude response to COVID-19 patients under the occurrence of negative physical and mental reactions. Considering that negative emotional responses such as fear and anger may be related to behavioral tendencies of the public toward the persons being infected with COVID-19, it is fundamental to investigate the factors influencing the public’s aversion responses and its resolutions. 

Therefore, in this context, based on stimulus–organism–response theory, this study aimed at exploring the relationships between environmental stimuli, public negative physiology, and aversion responses. Specifically, the present cross-sectional study was aimed at assessing: (1) the possible effects of the environmental stimuli including social media usage, risk communication, and prevention measures on the public’s negative physiology and public’s aversion to patients infected with COVID-19; (2) the role of negative physiology in linking the environmental stimuli and the public’s aversion of patients infected with COVID-19 in China. 

## 2. Theoretical Review

This part is focused on reviewing the literature related to the concepts and studies in constructing the theoretical framework of the research. The key concepts of the three stimuli variables and their possible relationships with negative physiology and public aversions are explained in details to help construct a complete theoretical framework. With reviewing the studies related to the stimuli and response variables, this part points out the gaps in existing research, and then proposes certain hypotheses and a conceptual model to be tested. 

### 2.1. Prevention Measures and Public Psychology

To prevent the large-scale spread of the epidemic, a series of prevention and control strategies were implemented around the world, including measures to limit population movement, maintain a safe social distance, and centralize or quarantine at home [2]. For example, at the beginning of the outbreak in Wuhan, China, in early 2020, the city was closed, including a complete shutdown of the public transportation system and a ban on the movement of people. After the outbreak was effectively controlled, the government implemented strict grid management measures and travel history tracking methods. After the outbreak became normalized, the public was required to comply with the outbreak prevention measures, wear masks, conduct temperature monitoring, and check registration information when entering public places.

Several studies have confirmed the effectiveness of the Chinese government’s prevention and control measures in controlling the spread of the outbreak. Lu et al. demonstrated that targeted outbreak prevention measures were effective in reducing the number of new cases in conjunction with government prevention measures in the three months following the outbreak of the COVID-19 pandemic [22]. Xu et al. show that the stronger the public perception of the effectiveness and fairness of the epidemic prevention and control system, the stronger their willingness to comply with the epidemic prevention measures [23]. Wu et al. showed that in a study of 1,049 residents, more than 80% of the residents were very understanding and supportive of various preventive measures such as closed management, strict travel restrictions, or mandatory wearing of masks in public [24]. 

However, at the same time, studies have also shown that prevention measures may have negative psychological effects on individuals. Hawryluck et al. showed that during the SARS epidemic, individuals experienced more pronounced PTSD symptoms the longer they were isolated [25]. This suggests that isolation itself, independent of knowledge of or exposure to SARS patients, may be individualized as traumatic. Lin et al. showed that both centralized and home isolation brought about high negative psychological effects, with age, area of residence, and history of exposure to novel coronavirus-infected persons being important factors influencing individuals’ physical and mental conditions [3]. Reynolds et al. demonstrated that the longer the individual felt isolated, the more pronounced the individual’s developed PTSD symptoms [26]. Xu et al., in a web-based survey of 2322 centrally and autonomously isolated individuals, found significantly higher levels of anxiety and depression in those isolated by COVID-19 [27]. Wang et al. showed that the depression level of the non-returned-to-work group was higher than that of the general population under the strict isolation measures of the COVID-19 pandemic and that individuals’ subjective perceptions of the severity of the epidemic positively affected their stress levels during the epidemic [28]. Li et al. found that public anxiety was more pronounced during home isolation and more pronounced if the public had a lower understanding of protective knowledge, stronger beliefs about preventive and control measures, and higher compliance with protective behaviors [29]. Based on the above literature review, this study proposes the following hypothesis.

**Hypothesis** **1** **(H1a).** *The perception of prevention measures significantly affects the public’s negative physiology during the epidemic*.

### 2.2. Social Media Usage and Risk Communication

Social media is a virtual network platform that provides a venue for people to share and exchange information, enabling users to communicate with each other based on interactive technology and realizing decentralized instant interaction, such as WeChat, Weibo, and so forth [30]. When a major public health emergency occurs, the public often has an urgent need for information. During this period, the public’s social media usage will also show certain special characteristics, such as length of use, attention, and increased access to information. 

In recent years, the value of the information generated by social media in the field of public health has been gaining attention [31]. Li et al. confirmed that among the media channels that influence the public’s preventive behavior, social media plays the most significant role, followed by news media [32]. Abdelhafiz et al. surveyed 559 urban and rural residents in 23 Egyptian governorates, noting that 66.9% of people chose to use social media to obtain information about the outbreak, much higher than the 35.4% who chose to communicate with health workers [33]. Of particular note is that Bhagavathula et al., in a study of 453 health care workers (HCWs) worldwide, noted that more than 61% of HCWs also primarily use social media to access relevant information [34]. 

Social media has become one of the most important channels for people around the world, including medical professionals, for obtaining information about the COVID-19 pandemic. In a study of Wuhan residents, Zhong et al. found that excessive use of social media during the epidemic led to mental health problems among respondents, including depression and secondary trauma [35]. Wang et al. showed that during the COVID-19 epidemic, moderate media dissemination of risk knowledge about the epidemic could lead to effective health literacy and thus motivate the public to adopt rational disease prevention behaviors. However, when the media overreports or distorts the risk, it is likely to cause more negative emotions in the public [36]. Based on the above literature review, this study proposes the following hypothesis.

**Hypothesis** **1** **(H1b).** *Social media usage significantly affects the public’s negative physiology during the epidemic*.

Risk communication refers to the process of sharing information between individuals and organizations [37]. In their survey, Wang et al. found that 93.5% of Chinese citizens used the Internet as their main channel for obtaining information related to the COVID-19 epidemic, and more than 53% of them believed that the epidemic had caused moderate or even severe negative psychological impact on them [4]. Risk information disseminated through the media or among people can significantly affect the public’s perception of their health risks [38]. In the absence of test tools or vaccines, social media overuse can cause damage to the public’s mental health. People who are heavily exposed to information related to the epidemic on social media are more likely to suffer from depression or secondary trauma than those who use social media less [35]. Person-to-person communication is also an important part of the daily dissemination of information about the risks of the disease, which can affect the public’s perception of the severity of the pandemic [39]. Based on this, this study proposes the following hypothesis.

**Hypothesis** **1** **(H1c).** *The perception of risk communication of COVID-19-related information significantly affects the public’s negative physiology during the epidemic*.

### 2.3. Negative Physiology and Public Aversion

Previous studies on natural disasters such as earthquakes and tsunamis have shown that while disasters cause direct damage to human life and property safety, they also have long-lasting effects on the psychological health of individuals [40]. An epidemic outbreak is a major public health emergency and a negative event that brings great uncertainty about virus infection and death. When individuals face public health emergencies, they pay excessive attention to information about the outbreak because of panic, which leads to biased risk perceptions of the seriousness of the threat of the outbreak, which in turn increases their depression and anxiety and other negative emotions [41]. 

Disgust is a negative emotional experience that is caused by an irritating stimulus [42]. The initial function of aversion is related to the defense against parasites and infections, and its role is to help humans defend themselves against disease-causing microorganisms. By identifying and moving away from disease-causing people or objects, humans can avoid being infected by pathogens, thus reducing the depletion of their immune system [43]. Faulkner et al. noted that individuals prefer to associate with in-group members and exclude contact with external group members to reduce the risk of pathogenic infections [7]. Based on the above literature review, this study proposes the following hypothesis.

**Hypothesis** **2** **(H2a).** *The public’s negative physiology during the epidemic arouses the public’s disgust toward patients infected with COVID-19*.

The concept of stigma was formally introduced in the 1960s by Goffman in the field of sociology and psychology, where he argued that stigma as a social state can cause the stigmatized person to lose his or her sense of reputation and worth. Through academic enrichment and development, stigma generally refers to the public’s negative and insulting description of a class of individuals or groups, and the discrimination and shunning of the stigmatized [44]. In the field of health research, stigma refers to the process of labeling, stereotyping, and discriminating against people who have a certain disease. A national online survey in Bangladesh reported that stigmatizing attitudes related to COVID-19 in the general population were significantly associated with marital status, education level, living conditions, and risk perception [9]. Research on public stigma has shown that individuals’ perceptions of stigma are related to cognitive attributions and emotional responses [45]. According to Weiner’s attribution theory, individuals are more likely to be stigmatized if they are perceived to have the ability to control the onset of their disease and to be responsible for their infections [46]. In addition to causal attributions of controllability and responsibility, individuals’ emotional responses such as fear and irritation are also associated with stigma [47]. This correlation has been found in many studies on the public stigma of mental illness, HIV/AIDS, and disability [6]. Within the framework of attribution theory and previous empirical studies, public stigma against patients with the COVID-19 epidemic may be associated with different attributions and adverse emotional responses. Given that the public stigma associated with the COVID-19 epidemic continues to escalate globally, it is important to investigate and address the public stigma associated with the COVID-19 epidemic in the general population. Based on this, this study proposes the following hypothesis.

**Hypothesis** **2** **(H2b).** *The public’s negative physiology during the epidemic arouses the public’s stigma toward patients infected with COVID-19*.

Avoidance is the process by which an individual is resisted and kept away by other organizations or individuals, causing the individual to lose his or her sense of belonging and linkage to other individuals in the group [48]. Social avoidance can severely affect the emotional state of the excluded person and have a significant impact on their physical and mental health. Using a study of 1803 rural older adults, Liu et al. confirmed that the stronger the perceived social avoidance, the stronger the negative emotional experience and that this experience significantly affects the self-health status of older adults [49]. Zhang et al. found that socially excluded individuals were more likely to seek risk and exhibit more risk-taking behavior when faced with risky decisions [50]. In a survey on social avoidance of mental illness, Wang found that respondents showed stronger avoidance of individuals with mental illness and that individuals would widen their social distance from the group and show a clear tendency to stigmatize [51]. Based on this, this study proposes the following hypothesis.

**Hypothesis** **2** **(H2c).** *The public’s negative physiology during the epidemic arouses the public’s avoidance of patients infected with COVID-19*.

The COVID-19 epidemic is full of risks and uncertainties, and the new characteristics and changing circumstances of the ultra-long incubation period and asymptomatic infected individuals have become important sources of negative public sentiment. Under the dual combination of unknown risk and fear of death, the public, dominated by fear and panic, disgust, stigma, and the avoidance of novel coronavirus-infected persons as a common behavioral response, became an important ground for the generation of public aversion. 

Based on this, this study proposes the following hypothesis:

**Hypothesis** **3** **(H3).** *The public’s negative physiology during the epidemic mediates the relationship between the environmental stimulus and the public’s aversion responses to patients infected with COVID-19*.

**Figure 1:**
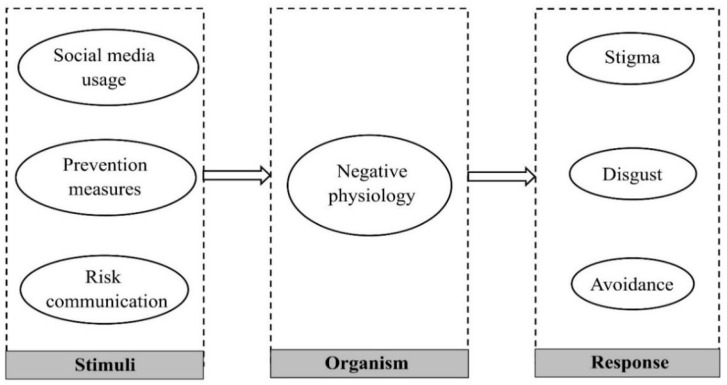
Conceptual model.

According to the hypotheses, the research model of this study is proposed as follows (Figure 1). 

## 3. Materials and Methods

**Figure 2:**
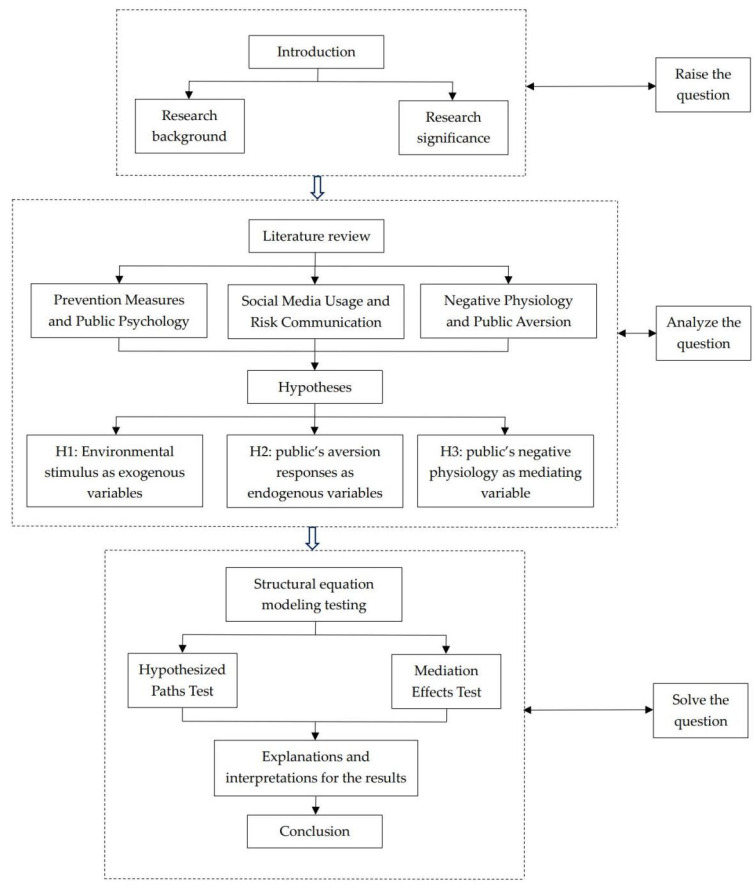
Steps of the research project.

A quantitative method was used in this study. Three hypotheses were proposed based on reviewing the literature related to the key concepts of this study. The statistical analyses were conducted on the base of the self-reported data collected through an online survey. The analysis results were discussed to generalize the perspectives that applied to certain conditional constraints and can be utilized in many situations. The steps of the research project are shown in Figure 2.

### 3.1. Measures

The measurement instruments used in this study were adapted from established scales. Social media usage during the epidemic was adapted from Zhong et al. [8]. Prevention measures, as well as negative physiology during the epidemic, were adapted from Lin et al. [3]. Risk communication was adapted from Cheng and Yin [52]. Stigma was adapted from Zhang et al. [53]. Disgust was adapted from Hodson [54]. Avoidance was adapted from Takeuchi [55]. 

### 3.2. Setting and Participants 

A cross-sectional, web-based survey was conducted on the sample service platform of Wenjuanxing (WJX) the largest Chinese online survey platform, in April 2022. The WJX sample service platform owns 6.2 million sample resources. The diversity of the sample resources on the WJX sample service platform is reflected in gender composition (48% females and 52% males), age distribution (21.04% for people below 20 years old, 25.03% for people between 21 and 25 years old, 29.34% for people between 26 and 30 years old, 16.26% for people between 31 and 40 years old, and 8.33% for people above 40 years old), identity structure (26.30% school students, 39.20% ordinary workers, 10.20% enterprise managers, 9.70% researchers, 4.20% civil servants, 3.10% professional and technical personnel, 1.80% freelancers, 5.50% others), and regional distribution (33.14% for people in northeastern cities in China, 51.71% for people in southeastern cities in China, 15.06 for people in western cities of China, 0.06% for people in Hong Kong and Macao, and 0.03% for people in other regions of China) [56]. 

The survey adopted random sampling to ensure that each respondent in the sample database had an equal chance of being selected. Each unit in the sample was completely independent, without specific correlation or exclusion between them. The link to the questionnaire was available to all the sample resources on WJX through both computers and smartphones. To avoid repetitive answers, it was set so that users with the same IP address and same computer/mobile phone equipment could only open the questionnaire one time. There was a disclaimer in the debriefing reminding the respondents that the study was a piece of academic research. We stressed the anonymity and privacy protection and encouraged participants to exit any time they felt uncomfortable. Each respondent received 5 RMB (about USD 80 cents) through the WJX system as incentive for participation. 

A pretest of the questionnaire’s applicability was administered before collecting the complete data; after collecting the first 50 questionnaires, the researcher suspended the release of the questionnaire and adjusted the questions based on the gained answers before releasing the final questionnaire. In the process, 1951 respondents joined and completed the survey. Respondents who chose the same number (e.g., chose 1) throughout the whole questionnaire were deleted to ensure the quality of the data [57]. A total of 1863 effective responses from participants of diverse demographics were received. In the Seventh National Census, China had a total population of 1.443 billion [58]; therefore, a sample of at least 1111 individuals was estimated for evaluating the selected variables, assuming a 3% level of precision (sampling error), and a response proportion of 50% with a 95% confidence level [59]. 

The collected sample was of diverse demographics (Table 1). In this sample, 73.1% of respondents were urban and 26.9% were rural, and the ratio of urban to rural population is about 7 to 3, which is relatively close to the current situation of urban and rural population distribution in China. According to China’s Seventh National Census released in 2021, 63.89% of the entire population lives in cities and towns, while 36.11% lives in the countryside [60]. Along with the process of urbanization in China, the urban population continues to rise. More than 80% of respondents of this sample were below 59 years old, in line with the 81.3% of the national population younger than 60 [54]. Meanwhile, according to a survey of Chinese people’s media use by *China Journalist*, sponsored by the Xinhua News Agency, social media is more popular among people under 30 in China [61]. This study focuses on the effects of social media usage and the risk communication through social media, together with the impacts of prevention and control measures, on the public’s aversion to patients infected with COVID-19. Therefore, in this sample, the proportion of respondents aged 18–30 is relatively large (37.7%), which is in line with China’s current national conditions and the status of social media use. 

## 4. Results and Analysis

### 4.1. Multivariate Normality and Common Method Biases Test

MPLUS 8.1 (TestPros, Sterling, Virginia, USA) and SPSS 22.0 (IBM, Armonk, NY, USA) were used to analyze the data collected in this study. Before the structural equation modeling analysis, SPSS was used to address missing values and test the multivariate normality and common method biases. 

Firstly, the missing values were processed. Through frequency analysis, the results showed that the proportion of missing values in the data was less than 5%, and the expectation–maximization algorithm was chosen to replace all the missing values. Secondly, descriptive statistics were carried out for all measurement items. As shown in Table 2, the skewness of all but three indicators was between +1 and −1. Additionally, 43 of the 52 indices had kurtosis between +1 and −1. This means that 43 items followed a normal distribution. Therefore, the data in this study did not deviate from the normal distribution requirement [62]. Finally, Harman’s single-factor test was used to conduct the common method biases test for the data [63]. All the measured items were included in the factor analysis. The results showed that the variance explanation rate of the first factor was 37.162% (<40%), there were 5 (>2) factors with eigenvalues greater than 1, and together, they explained 71.316% of the variance. This indicates that the data did not suffer from common method biases.

### 4.2. Constructs Measurement

We evaluated the reliability and validity of the seven first-order constructs in this part. The 8 items with factor loadings less than 0.6 were deleted, and the factor loadings of the remaining 44 items were above the recommended level of 0.6 [64]. The Cronbach’s α ranged from 0.712 to 0.944, which was higher than the recommended value of 0.6 [65]. The construct reliability (CR) ranged from 0.714 to 0.948, which was higher than the recommended value of 0.7. The average variance extracted (AVE) was between 0.502 and 0.696, which is greater than the recommended threshold of 0.5. The results indicated that the measurement model has good internal consistency and convergent validity.

Then, confirmatory factor analysis (CFA) was performed on the overall measurement model after item deletion. The results showed that the ratio of chi-squared to degrees of freedom (CMIN/DF) was 2.210 (less than 5). The goodness-of-fit index (GFI) was 0.916, the value-added adaptation index (IFI) was 0.932, the comparison fitting index (CFI) was 0.932, and the root-mean- square error of approximation (RMSEA) was 0.057 (less than 0.08), indicating that the model fit the data well.

### 4.3. Hypothesized Paths Test

Inter-correlations for all pairs of constructs were first tested in SPSS 22.0 to check the relationships among the variables. The results in Table 3 showed that the three stimulus variables were all significantly correlated to negative physiology, and the three response variables were all significantly correlated to negative physiology, while not all three response variables were significantly correlated to the three stimulus variables. 

Inter-correlations were significant for all pairs of constructs (*p* < 0.05) except for the correlation between social media usage and prevention measures, which were assumed not to directly affect each other in the proposed model.

MPLUS 8.0 was used to conduct two-step structural equation model testing of the overall path of the proposed model. 

In the first step of the measurement phase, the work analyzed 44 measurement items and examined the correlated residuals and cross-loadings for each item to confirm that they could be combined into indices following the original measurement scales. 

In the second step, the confirmatory structural equation model was used to test the relationships among variables. The resulting model had good fit: CMIN/DF = 2.227; RMSEA = 0.064; CFI = 0.917; IFI = 0.917; GFI = 0.904, in accordance with Bagozzi and Yi [66]. 

**Figure 3:**
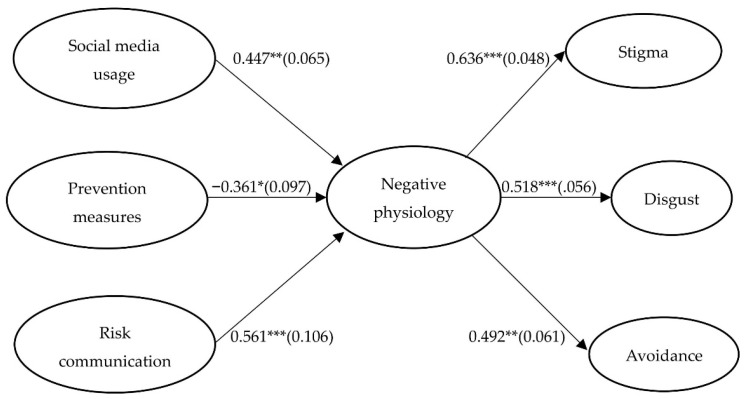
Statistical model. Note. * *p* < 0.05, ** *p* < 0.01, *** *p* < 0.001.

As shown in Figure 3, both social media usage (β = 0.447, *p* = 0.003) and perception of risk communication of COVID-19-related information (β = 0.561, *p* = 0.000) enhanced the public’s negative physiology during the epidemic, while the perception of prevention measures reduced the public’s negative physiology during the epidemic (β = −0.361, *p* = 0.014). Thus, H1 was supported that environmental stimuli significantly affected the public’s negative physiology during the epidemic. 

Meanwhile, the public’s negative physiology during the epidemic positively affected the public’s aversion responses to people being infected, including stigma (β = 0.636, *p* = 0.000), disgust (β = 0.518, *p* = 0.000), and avoidance (β = 0.492, *p* = 0.001<0.01). Thus, H2 was supported that the public’s negative physiology during the epidemic aroused the public’s aversion responses to patients infected with COVID-19.

In addition, in terms of the direct relationships between these three stimuli variables and these three response variables, there were no direct relationships between social media usage and the response variables (disgust (β = 0.023, *p* = 0.664 > 0.05), avoidance (β = 0.015, *p* = 0.871 > 0.05)) except for stigma (β = 0.103, *p* = 0.032 < 0.05); there were significant direct relationships between prevention measures and these three response variables (stigma (β = −0.124, *p* = 0.016 < 0.05), disgust (β = −0.096, *p* = 0.034 < 0.05), avoidance (β = −0.115, *p* = 0.027 < 0.05)); and there were significant direct relationships between risk communication and two of the response variables (disgust (β = 0.132, *p* = 0.018 < 0.05), stigma (β = 0.109, *p* = 0.021 < 0.05)) but not avoidance (β = 0.053, *p* = 0.062 > 0.05). 

Through the above data analysis, it was found that these three stimulus variables and these three response variables were separately significant exogenous variables and endogenous variables of the public’s negative physiology. However, not all three of the stimulus variables significantly affected the three response variables. The following tests examined the mediation effects of negative physiology between the three stimulus and three response variables. 

### 4.4. Mediation Effects Test

Using the bootstrap syntax, model indirect, and cinterval directives in MPLUS [67], the direct relationships between the three stimulus variables and three response variables were controlled. Table 4 shows that the paths from the environmental stimuli, including social media usage [95%CI(0.297, 0.443)], perception of prevention measures [95%CI(−0.299, −0.057)], and perception of risk communication of COVID-19-related information [95%CI(0.111, 0.197)], to the public’s stigma of people being infected via the public’s negative physiology did not contain zero. 

Meanwhile, the paths from the environmental stimulus, including social media usage [95%CI(0.329, 0.567)], perception of prevention measures [95%CI(−0.307, −0.079)], and perception of risk communication of COVID-19-related information [95%CI(0.146, 0.294)], to the public’s disgust of people being infected via the public’s negative physiology did not contain zero. In addition, the paths from the environmental stimuli, including social media usage [95%CI(0.183, 0.409)], perception of prevention measures [95%CI(−0.284, −0.046)], and perception of risk communication of COVID-19-related information [95%CI(0.127, 0.283)], to the public’s avoidance of people being infected via the public’s negative physiology did not contain zero. 

These results supported H3 that the public’s negative physiology during the epidemic mediated the relationships between the environmental stimuli and the public’s aversion responses to patients infected with COVID-19. 

## 5. Discussion

This research aimed to understand the factors influencing the public’s aversion to patients infected with COVID-19 in China. The results show that environmental factors, including the use of social media, realistic prevention measures, and risk communication, all significantly affected the negative physiology of the public, in turn intensifying the public’s aversion, including aversion, stigma, and rejection, of people being infected.

### 5.1. Scientific and Rational Risk Communication during the Epidemic

In a public health emergency, the public has a stronger demand for information and can quickly accept and disseminate risk information through social media [68]. The spread of risk information affected the negative physiology of the public. During COVID-19, risk communication has not been limited to the media reality but also exists in the environment of interpersonal communication in the real space [9]. By learning more information about the epidemic, the public can enhance their understanding of the development of the epidemic and social stability. However, when the risk awareness is too strong, the public is prone to panic [69].

Under the control of negative emotions such as fear and panic, the stigmatization of infected people became a common behavioral response. Nearly 80% of individuals tended to maintain social distancing with patients infected with COVID-19 [70]. In the context of COVID-19, both the media reality and the real environment are giving hints about the risks of the epidemic. Out of the fear of uncertainty and the anxiety of close contact with illness and death, people will choose to acquire as much information as possible to improve their sense of security. However, exposure to excessive negative information will amplify individuals’ perceptions of current risks. In the face of risky events and the negative impacts they may bring, reducing contact with and even stigmatizing people being infected is likely to be a way for the public to avoid risks and enhance their sense of security.

When public health emergencies occur, the formulation and improvement of health policies are of great significance. The lack of scientific knowledge of the epidemic may increase the public’s discrimination against patients infected with COVID-19 [44]. Governments as well as the WHO should develop and improve policies aimed at reducing stigma and, through scientific dissemination, guide the public to a correct understanding of COVID-19. Anti-stigmatization should be the focus of public health policy so that the public has a healthier perception and attitude towards those infected with diseases such as COVID-19. The improvement of the scientific cognition of diseases can reduce the irrational and negative emotions of the public and help maintain the benign social order. The prevention and control experiences during the 2013 SARS period in China proved that if individuals have a relatively scientific understanding of the epidemic, they will make more rational behavioral responses based on a sense of security [71].

### 5.2. Strict Gate-Keeping of Information Related to the Epidemic on Social Media

During the COVID-19 pandemic, especially during the large-scale containment and quarantine, the public was used to sharing their personal emotions, especially negative emotions, on social media platforms [72]. The public’s negative physiology during the epidemic mediated the relationships between the environmental stimul and the public’s aversion responses to patients infected with COVID-19. Improper attribution and negative physiology are the causes of irrational behaviors such as stigmatization and rejection [17]. Misconceptions and lack of knowledge about novel coronavirus, as well as high levels of fear, are associated with stigmatized attitudes towards people closely related to novel coronaviruses, such as health care workers and infected people [73]. The spread of the epidemic posed a threat to the public’s mental state, combined with reports of people being labeled with epidemic infection and risk communication spreading in great quantities in social media and reality, the public’s anxiety, depression, and other psychological distress are caused, and in turn, these bad psychological conditions influenced the public’s tendency to reject infected persons.

To deal with this problem, the government should timely disclose information about the epidemic to protect citizens’ right to know, and media practitioners should always adhere to the transmission of true and objective information and guide the public to understand the epidemic rationally. 

The government should pay attention to continuous, timely, and appropriate risk communication with the help of social media, strictly gatekeeping information related to the outbreak posted on social media, to effectively manage and control the negative public opinion during the epidemic [74].The government can regulate the level of government trust and public risk perception through multimedia risk communication, reduce the impact of inaccurate news on social media on the public, and mitigate the negative psychological impact on the public during the epidemic. This not only protects the personal interests of infected persons but also promotes public compliance with government policies.

### 5.3. Prevention and Protection of Individual Life during the Epidemic

The public’s perception of prevention and control measures during the epidemic has played an important role in reducing the public’s negative physiology. Since the outbreak of COVID-19, various prevention and control measures have been adopted by the Chinese government, including home quarantine, social distancing, and registration in public places. These measures have effectively curbed the spread of the epidemic and are in line with the needs of individuals in China, a population superpower, in seeking health and safety protection. Individual compliance with quarantine prevention and control measures was negatively correlated with their anxiety levels [24]. That is, the stricter the prevention and control measures, the lower the incidence of negative emotions [75]. Appropriate measures to implement humanized prevention and control measures may enhance the sense of security of the public towards their living environment and in turn help people adjust to the negative physiology of individuals during the epidemic.

In the process of epidemic prevention and control, the public’s aversion to novel coronavirus infected persons has led to cases where some basic rights of novel coronavirus infected persons cannot be guaranteed in social life, and their interests are damaged. For example, persons with novel coronavirus infections have been flirted with, harassed, or verbally abused by netizens after their personal information and travel trails have been made public. Government staff spread the personal information of novel coronavirus-infected persons they learned about in the course of their work freely, causing serious distress to the people involved [76]. This requires the government to strike a balance between the public’s right to know and the patient’s right to privacy in the epidemic prevention and control process. This means that not only should information about the epidemic be made public, the root cause of the epidemic be found in the first place, and the spread of the epidemic be effectively blocked but also the privacy rights of novel coronavirus infected persons need to be protected.

### 5.4. Limitations and Suggestions for Future Research

There are several limitations of this study. First, the use of an unreliable instrument may limit the rigor and universality of the findings, and the cross-sectional survey may lead to uneven population coverage and selection bias. In future studies, it may be considered to use more standard tools for measuring the variables and expand the coverage of the sample population or adopt more stringent sampling methods to improve the diversity and representativeness of the sample population distribution. 

Second, this research regarded the public’s negative physiology as an overall variable without separately testing the different dimensions (sadness, boredom, isolation, frustration, and so forth) of negative physiology in terms of their different effects on the behavioral tendencies of the public. 

Third, the environmental impacts brought by the COVID-19 epidemic are reflected in the social media platform, the dissemination of risk information in the virtual environment, and the prevention measures and atmosphere in the real environment, but at the same time, the stage performance, period and specific location of the epidemic may also have impacts on the public’s negative emotions and aversion psychology, and the dynamic tracking approach can be tried in future studies to compare. Future studies may explore the dynamic changes in public psychology during major public health emergencies by comparing the changes in variables during different periods and stages of the epidemic. 

Fourth, the current study was conducted in the Chinese society and reported Chinese peoples’ responses, which limits the external validity of this study. However, given the commonalities of human nature in terms of cognitive and emotional activities during the epidemic situation, the authors believe that the path proved in this study is also applicable to people in other cultural contexts without seriously disturbing the external validity of the findings. Future comparative and replicate studies that examine the application of the proposed path cross-nationally and cross-culturally may deliver new academic and managerial insights for guiding public psychology during the pandemic. 

Lastly, studies on individuals involve complex social psychology and behavior, and more generalized conclusions may be achieved if other approaches can be combined to record and track people’s specific behaviors and psychological states over time and explore the causal mechanisms between them. 

## 6. Conclusions

This research highlights the nonnegligible effects of the daily use of social media and the risk communication of COVID-19-related information in aggravating the public’s negative physiology, which in turn enhances the public’s resistance and disgust to the patients being infected. Strict prevention and control measures may play an important role in strengthening the public’s physical and mental security and reducing the public’s aversion to patients infected with COVID-19 in the context of Chinese society.

With the continuous rebound of the global COVID-19 epidemic, all countries still need to make full preparations for prevention and control. Appropriate and effective prevention and control measures can meet the safety boundary of individual psychology and enhance the public’s trust in the social environment. When formulating prevention and control measures, full consideration should be given to the personal physical and mental conditions of residents to achieve the greatest degree of humanized management. At the same time, during the prevention and control period, it is also necessary to disclose official information and respond to public demands promptly to alleviate public tension, panic, and other emotions and help eliminate public stigma appropriately and scientifically.

## Figures and Tables

**Table 1:** Participant characteristics (N = 1863).

n%			n%		
Age Group (yrs)			Occupation Type		
<18	15	0.8	Farmers	81	4.3
18–29	702	37.7	Health professionals	173	9.3
30–39	417	22.4	Students	494	26.5
40–49	325	17.4	Officers	523	28.1
50–59	150	8.1	Industrial workers	257	13.8
>59	254	13.6	Self-employed person	40	2.1
**Gender**			Others	295	15.8
Male	882	47.3	**Educational level**		
Female	981	52.7	Secondary school and below	638	34.2
**Average monthly income (RMB)**			High school	661	35.5
<2000	736	39.5	University	564	30.3
2001–5000	427	22.9	**Location of current workplace**		
5001–10,000	503	27.0	Urban	1362	73.1
>10,000	197	10.6	Rural	501	26.9

**Table 2:** Assessment of the items and variables (N = 1863).

*Social Media Usage:*	Skewness	Kurtosis	SFL
Cronbach’s α = 0.906; CR = 0.905; AVE = 0.615			
1. I spent a lot of time thinking about the COVID-19 content on WeChat.	−0.537	0.368	0.702
2. I want to use WeChat more to learn about COVID-19.	−0.476	0.435	0.689
3. I have been following COVID-19 content on WeChat to ease worries about daily life.	−0.872	−0.754	0.676
4. I tried to reduce the frequency of using WeChat to learn about COVID-19 but without success. *	−0.682	1.262	0.595
5. I would be troubled if I was banned from using WeChat to get information about COVID-19.	−0.987	0.563	0.698
6. Paying attention to information about COVID-19 on WeChat too often has some negative effects on my life.	−0.343	0.122	0.643
***Prevention measures:***			
Cronbach’s α = 0.937; CR = 0.940; AVE = 0.636			
7. Use separate towels	−0.834	0.643	0.657
8. Wash hands frequently	−0.643	0.427	0.746
9. Use separate cutlery	−0.564	0.993	0.703
10. Sleep in separate rooms	−0.911	0.754	0.689
11. Wear a mask on all occasions	−0.654	0.219	0.767
12. Compliant with all household prevention measures	−0.766	0.315	0.728
13. Do not go out of the house to socialize	−0.544	0.536	0.732
14. Do not attend important events	−0.348	0.369	0.745
15. Do not allow visitors into the home	−0.175	0.743	0.71
16. Compliant with all community protective measures	−0.765	1.572	0.724
***Risk communication:***			
Cronbach’s α = 0.838; CR = 0.839; AVE = 0.597			
17. Friends close to me say it is very dangerous to get COVID-19.	−0.623	0.264	0.689
18. I have seen frequent media coverage of COVID-19 risks.	0.347	0.854	0.725
19. The risk information of COVID-19 is often circulated in my community (or wechat group).	−0.436	0.643	0.717
***Negative physiology:***			
Cronbach’s α = 0.944; CR = 0.948; AVE = 0.696			
20. perception of boredom	−0.762	0.352	0.709
21. perception of isolation	−0.434	1.056	0.735
22. perception of frustration	−0.452	0.985	0.688
23. perception of annoyance	−0.562	0.346	0.645
24. perception of worry	−0.786	0.762	0.721
25. perception of loneliness	−0.923	0.851	0.733
26. perception of helpless	−0.658	0.363	0.714
27. perception of anger	−0.348	0.738	0.693
28. perception of nervousness	−0.873	0.564	0.722
29. perception of sadness	−0.345	−0.373	0.758
***Stigma:***			
Cronbach’s α = 0.808; CR = 0.811; AVE = 0.502			
30. Patients infected with COVID-19 are, in a way, repulsive. *	0.965	2.632	0.556
31. I fear that the people being infected may cause harm to others. *	1.194	1.735	0.544
32. I will try to keep a distance from people being infected.	−0.763	0.747	0.636
33. People being infected can be troublesome. *	−0.887	0.962	0.598
34. People being infected have increased the pressure on social governance.	−0.374	0.371	0.606
35. When I know of someone being infected, I try to stay away from him/her.	0.478	0.262	0.641
36. People being infected cause inconvenience to the daily lives of others.	−0.369	0.743	0.659
37. I’m afraid of being alone with a person being infected.	−0.863	0.632	0.707
38. People being infected are understandably ostracized and alienated by others.	−0.473	0.367	0.612
39. When I meet people who have been to the affected areas, I try to keep them at arm’s length.	−0.84	0.744	0.694
***Disgust:***			
Cronbach’s α = 0.712; CR = 0.714; AVE = 0.598			
40. I feel sick when someone infected with COVID-19 invades my personal space. *	−0.983	1.073	0.587
41. I wash my hands after shaking hands with someone who is infected, even if his/her hands are clean.	0.493	0.463	0.711
42. I would hate to be in a confined space such as an elevator where someone being infected has stayed, even if the space has been disinfected.	−0.839	0.637	0.686
43. If I were told that I was the spacetime companion of someone being infected, I might be alienated from healthy people.	−0.495	0.983	0.604
44. I would be concerned if I had been in face-to-face contact with someone being infected.	−0.874	0.463	0.736
45. If my cook turns out to be infected, I would be disturbed. *	−1.263	2.254	0.523
***Avoidance:***			
Cronbach’s α = 0.839; CR = 0.843; AVE = 0.532			
46. I will keep a physical distance from those infected with COVID-19.	0.841	0.036	0.643
47. If any of my colleagues are infected with novel Coronavirus, I will try to avoid them. *	−0.943	1.986	0.576
48. I will try to avoid people being infected. *	1.073	1.542	0.552
49. I would take offense at some of the behavior of people being infected.	0.368	0.357	0.602
50. I would be annoyed by some of the behavior of people being infected.	0.763	0.263	0.612
51. I would find it very difficult to be in close contact with someone being infected.	−0.538	0.643	0.657
52. I would feel overwhelmed if I had to talk to someone being infected.	−0.726	0.821	0.635

**Note**: Alpha refers to Cronbach’s alpha reliability estimate; CR refers to composite reliability estimate; AVE refers to average variance extracted; SFL refers to standardized factor loading. * indicates items deleted in the model test.

**Table 3:** Bivariate Correlations Estimates.

Variable	1	2	3	4	5	6	7
1. Social media usage	/						
2. Prevention measures	0.092	/					
3. Risk communication	0.650 ***	0.157 *	/				
4. Negative physiology	0.530 ***	−0.497 ***	0.602 ***	/			
5. Stigma	0.197 *	−0.167 *	0.183 *	0.74 ***	/		
6. Disgust	0.087	−0.139 *	0.167 *	0.652 ***	0.510 **	/	
7. Avoidance	0.079	−0.161 *	0.075	0.603 ***	0.266 **	0.312 **	/

**Note:** * *p* < 0.05, ** *p* < 0.01, *** *p* < 0.001.

**Table 4:** Confidence intervals of standardized mediation effects.

	*L* 0.5%	*L* 2.5%	*L* 5%	Estimate	*U* 5%	*U* 2.5%	*U* 0.5%
SMU to ST via NP	0.281	0.297	0.313	0.370	0.427	0.443	0.459
SMU to DI via NP	0.297	0.329	0.365	0.448	0.531	0.567	0.599
SMU to AV via NP	0.164	0.183	0.225	0.296	0.367	0.409	0.428
PCM to ST via NP	−0.353	−0.299	−0.240	−0.178	−0.116	−0.057	−0.003
PCM to DI via NP	−0.379	−0.307	−0.252	−0.193	−0.134	−0.079	−0.007
PCM to AV via NP	−0.328	−0.284	−0.222	−0.165	−0.108	−0.046	−0.002
RC to ST via NP	0.098	0.111	0.130	0.154	0.178	0.197	0.210
RC to DI via NP	0.104	0.146	0.173	0.220	0.267	0.294	0.336
RC to AV via NP	0.106	0.127	0.151	0.205	0.259	0.283	0.304

**Note.** SMU = Social media usage, PCM = Prevention measures, RC = Risk communication, NP = Negative physiology, ST = Stigma, DI = Disgust, AV = Avoidance, L = Lower, and U = Upper.

## Data Availability

The data that support the findings of this study are available from the first author, K.Z., upon reasonable request.

## References

[1] Coronavirus Research Center, Johns Hopkins University & Medicine (2022). COVID-19 Dashboard by the Center for Systems Science and Engineering (CSSE) at Johns Hopkins University (JHU). https://coronavirus.jhu.edu/map/.

[2] Chinazzi M., Davis J.T., Ajelli M., Gioannini C., Litvinova M., Merler S., Piontti Y., Pastore A., Mu K., Rossi L. (2020). The effect of travel restrictions on the spread of the 2019 novel coronavirus (COVID-19) outbreak. Science.

[3] Lin Y., Hu Z., Alias H., Wong L.P. (2021). Quarantine for the coronavirus disease (COVID-19) in Wuhan city: Support, understanding, compliance and psychological impact among lay public. J. Psychosom. Res..

[4] Wang C., Pan R., Wan X., Tan Y., Ho R.C. (2020). Immediate psychological responses and associated factors during the initial stage of the 2019 coronavirus disease (COVID-19) epidemic among the general population in China. Int. J. Environ. Res. Public Health.

[5] Corrigan P.W., Watson A.C., Warpinski A.C., Gracia G. (2004). Stigmatizing attitudes about mental illness and allocation of resources to mental health services. Community Ment. Health J..

[6] Corrigan P.W., Markowitz F.E., Watson A.C., Kubiak R.M.A. (2003). An attribution model of public discrimination towards persons with mental illness. J. Health Soc. Behav..

[7] Faulkner J., Schaller M., Park J.H., Duncan L.A. (2004). Evolved disease-avoidance mechanisms and contemporary xenophobic attitudes. Group Processes Intergroup Relat..

[8] Shultz J.M., Baingana F., Neria Y. (2015). The 2014 Ebola outbreak and mental health: Current status and recommended response. J. Am. Med. Assoc..

[9] Hossain M.B., Alam M., Islam M., Sultan S., Faysal M., Rima S., Hossain M.A., Mahmood M.M., Kashfi S.S., Mamun A.A. (2021). COVID-19 public stigma in the context of government-based structural stigma: A cross-sectional online survey of adults in Bangladesh. Stigma Health.

[10] Mehrabian A., Russell J.A. (1974). An Approach to Environmental Psychology.

[11] Wang Y., Wang T., Liu Z., Li J. (2020). A study on citizen health emergency behavior based on SOR model. Chongqing Soc. Sci..

[12] Pan T., Lu Y. (2022). Research on influencing factors of user participation behavior based on SOR model in online health community. Inf. Doc. Serv..

[13] Wang Z., Xu H., Zheng L., Liu X. (2021). Willingness for primary diagnosis and influencing factors among residents in Jiangsu based on SOR theory. Mod. Prev. Med..

[14] Lei J., Jiang G. (2018). An empirical study on customer’s acceptance willingness to opaque sales of perishable good. J. Tech. Econ. Manag..

[15] Xiang Y.T., Yang Y., Li W., Zhang L., Zhang Q., Cheung T., Ng C.H. (2020). Timely mental health care for the 2019 novel coronavirus outbreak is urgently needed. Lancet Psychiatry.

[16] Bao Y., Sun Y., Meng S., Shi J., Lu L. (2020). 2019-nCoV epidemic: Address mental health care to empower society. Lancet.

[17] Zhang J., Lu H., Zeng H., Zhang S., Du Q., Jiang T., Du B. (2020). The differential psychological distress of populations affected by the COVID-19 pandemic. Brain Behav. Immun..

[18] Daly M., Robinson E. (2021). Psychological distress and adaptation to the covid-19 crisis in the united states. J. Psychiatr. Res..

[19] Gallè F., Sabella E.A., Roma P., Ferracuti S., Da Molin G., Diella G., Montagna M.T., Orsi G.B., Liguori G., Napoli C. (2021). Knowledge and lifestyle behaviors related to covid-19 pandemic in people over 65 years old from southern italy. Int. J. Environ. Res. Public Health.

[20] Brooks S.K., Webster R.K., Smith L.E., Woodland L., Wessely S., Greenberg N., Rubin G.J. (2020). The psychological impact of quarantine and how to reduce it: Rapid review of the evidence. Lancet.

[21] Qiu J., Shen B., Zhao M., Wang Z., Xie B., Xu Y. (2020). A nationwide survey of psychological distress among Chinese people in the COVID-19 epidemic: Implications and policy recommendations. Gen. Psychiatry.

[22] Lu X., Bian Y. (2021). The two forces for epidemic risk governance—Government prevention and control measures and online public participation. Jiangsu Soc. Sci..

[23] Xu B., Zhang S., Guo H., Zhu A. (2020). Research on the mechanism behind the influence of the degree of public epidemic prevention and control perceptions on compliance behavior—theoretical and empirical analyses based on COVID-19. China Public Adm. Rev..

[24] Wu Y., Chen Z., Chen J. (2021). Historical Memory and Resident’s Suffering Consciousnes: The LongTerm Impact of SARS on Preventing COVID-19. Rev. Ind. Econ..

[25] Hawryluck L., Gold W.L., Robinson S., Pogorski S., Galea S., Styra R. (2004). SARS control and psychological effects of quarantine, Toronto, Canada. Emerg. Infect. Dis..

[26] Reynolds D.L., Garay J.R., Deamond S.L., Moran M.K., Gold W., Styra R. (2008). Understanding, compliance and psychological impact of the SARS quarantine experience. Epidemiol. Infect..

[27] Xu C., Mo K., Liu X., Lu W., Xu X., Peng L., Li M. (2020). Survey of anxiety and depressive symptoms and analysis of their risks factors among individuals quarantined for COVID-19. J. Third Mil. Med. Univ..

[28] Wang Z., Liu W., Lou S., Ma X., Zahng X., Guo H., Ma C., Chen W., Yang Y. (2021). Status and factors of pressure/depression in common population under quarantine measures of COVID-19 pandemic. China J. Health Psychol..

[29] Li J., Wang S., Yu R., Hu J., Jiang M., Huang W. (2020). Compliance of home quarantine protection and its effect on anxiety degree during the epidemic outbreak period of COVID-19. Chin. J. Infect. Control..

[30] Obar J.A., Wildman S. (2015). Social media definition and the governance challenge: An introduction to the special issue. Telecommun. Policy.

[31] Rivers C., Chretien J.P., Riley S., Pavlin J.A., Woodward A., Brett-Major D., Berry I.M., Morton L., Jarman R.G., Biggerstaff M. (2019). Using “outbreak science” to strengthen the use of models during epidemics. Nature Communications.

[32] Li X., Liu Q. (2020). Social media use, ehealth literacy, disease knowledge, and preventive behaviors in the COVID-19 pandemic: Cross-sectional study on Chinese netizens. J. Med. Internet Res..

[33] Abdelhafiz A.S., Mohammed Z., Ibrahim M.E., Ziady H.H., Alorabi M., Ayyad M., Sultan E.A. (2020). Knowledge, perceptions, and attitude of egyptians towards the novel coronavirus disease (COVID-19). J. Community Health.

[34] Bhagavathula A.S., Aldhaleei W.A., Rahmani J., Mahabadi M.A., Bandari D.K. (2020). Novel coronavirus (COVID-19) knowledge and perceptions: A survey of healthcare workers (preprint). JMIR Public Health Surveill..

[35] Zhong B., Huang Y., Liu Q. (2021). Mental health toll from the coronavirus: Social media usage reveals Wuhan residents’ depression and secondary trauma in the COVID-19 outbreak. Comput. Hum. Behav..

[36] Wang Y., Gao J., Chen H., Mao Y., Chen S., Dai J., Zheng P., Fu H. (2020). The relationship between media exposure and mental health problems during COVID-19 outbreak. Fudan Univ. J. Med. Sci..

[37] Leiss W. (1996). Three phases in the evolution of risk communication practice. Ann. Am. Acad. Political Soc. Sci..

[38] Li X., Liu Q. (2021). Risk propagation expansion model from the perspective of media credibility: Based on a national survey of COVID-19. Mod. Commun..

[39] Xie Q. (2021). The influence of media use and panic on social media users’ curatorial news of epidemic. Chin. J. Journal. Commun..

[40] Li X., Wu X. (2021). Different depression in one city: Gender, class and mental health disparities during COVID-19 in Wuhan. Popul. Dev..

[41] Choi D.H., Yoo W., Noh G.Y., Park K. (2017). The impact of social media on risk perceptions during the MERS outbreak in South Korea. Comput. Hum. Behav..

[42] Jin Y., Zhang D., Liu J., Luo Y. (2014). An erp study of disgust processing. Acta Psychol. Sin..

[43] Peng M., Zhang L. (2016). How disgust affects moral judgment across age groups. J. Psychol. Sci..

[44] Miao D., Xia M. (2021). Study of stigma in risk society—A exploratory research based on the stigma of “Hubei people” During the COVID-19. J. China Agric. Univ. (Soc. Sci.).

[45] Corrigan P.W. (2000). Mental health stigma as social attribution: Implications for research methods and attitude change. Clin. Psychol. Sci. Pract..

[46] Weiner B. (1993). On sin versus sickness. A theory of perceived responsibility and social motivation. Am. Psychol..

[47] Corrigan P.W., River L.P., Lundin R.K., Wasowski K.U., Campion J., Mathisen J., Goldstein H., Bergman M., Gagnon C., Kubiak M.A. (2000). Stigmatizing attributes about mental illness. J. Community Psychol..

[48] Du J., Xia B. (2008). The Psychological View on Social Exclusion. Adv. Psychol. Sci..

[49] Liu C., Dong Y., Wang H., Zhang D. (2021). Social Exclusion Experience and Health Condition among the Rural Elders: The Moderated Mediating Effect. Psychol. Dev. Educ..

[50] Zhang S., Huang J., Zhao F., Xu K. (2022). Social exclusion influenced intertemporal decision-making and its mechanism. Adv. Psychol. Sci..

[51] Wang X. (2019). Social exclusion of mental illness and its influence on doctor-patient relationship: A study based on chinese general social survey. J. Nanjing Norm. Univ. (Soc. Sci. Ed.).

[52] Cheng Y., Yin J. (2021). Has COVID-19 increased the intention to undertake health tourism? Examination using a conditional process Model. Tour. Trib..

[53] Zhang T.M., Fang Q., Yao H., Mao R.S. (2021). Public stigma of COVID-19 and its correlates in the general population of China. Int. J. Environ. Res. Public Health.

[54] Hodson G., Choma B.L., Boisvert J., Hafer C.L., Macinnis C.C., Costello K. (2013). The role of intergroup disgust in predicting negative outgroup evaluations. J. Exp. Soc. Psychol..

[55] Takeuchi E., Fujisawa D., Miyawaki R., Yako-Suketomo H., Oka K., Mimura M., Takahashi M. (2020). Cross-cultural validation of the cancer stigma scale in the general Japanese population. Palliat. Supportive Care.

[56] WJX Sample Source and Sample Composition. https://www.wjx.cn/sample/service.aspx.

[57] Meade A.W., Craig S.B. (2012). Identifying careless responses in survey data. Psychol. Methods.

[58] National Bureau of Statistics Seventh National Census Bulletin. www.stats.gov.cn/tjsj/tjgb/rkpcgb/qgrkpcgb/202106/t20210628_1818821.html.

[59] Israel G.D. (1992). Determining Sample Size.

[60] National Bureau of Statistics (2021). Bulletin of the Seventh National Census. www.gov.cn/guoqing/2021-05/13/content_5606149.htm.

[61] Li L., Liang Y., Dai L. (2022). Report of questionnaire survey on media use among young people. Chin. J..

[62] Kline R.B. (1998). Principles and practice of structural equation modeling. J. Am. Stat. Assoc..

[63] Zhang H., Wu Y., Buhalis D. (2017). A model of perceived image, memorable tourism experiences and revisit intention. J. Destin. Mark. Manag..

[64] Hu L.T., Bentler P.M. (1999). Cutoff criteria for fit indexes in covariance structure analysis: Conventional criteria versus new alternatives. Struct. Equ. Modeling.

[65] Petrick J.F., Backman S.J. (2002). An examination of the construct of perceived value for the prediction of golf travelers’ intentions to revisit. J. Travel Res..

[66] Bagozzi R.P., Yi Y. (2012). Specification, evaluation, and interpretation of structural equation models. J. Acad. Mark. Sci..

[67] Muthén L.K., Muthén B.O. (2017). Mplus User’s Guide.

[68] Miles B., Morse S. (2007). The role of news media in natural disaster risk and recovery. Ecol. Econ..

[69] Agha S. (2003). The impact of a mass media campaign on personal risk perception, perceived self-efficacy and on other behavioural predictors. AIDS Care.

[70] Lee S., Lee S., Fung C.S., Kwok K.P. (2008). Public attitudes toward SARS and their implications for societal preparedness for other emerging infections. Soc. Med..

[71] Zhao Y. (2003). Social discrimination during SARS and its causes. Youth Stud..

[72] Ren W., Zhu X., Hu Y. (2021). Risk perception and preventive behaviors: A comparison of the multifaceted effects of social media and authoritative media during the outbreak of COVID-19. Chin. J. Journal. Commun..

[73] Dhanani L.Y., Franz B. (2020). Unexpected public health consequences of the COVID-19 pandemic: A national survey examining anti-Asian attitudes in the USA. Int. J. Environ. Res. Public Health.

[74] Lyu J., Wang W., Zhang Y., Zhou J., Wang S. (2020). Research progress of social media in coronavirus disease 2019 (COVID-19). Chin. J. Dis. Control. Prev..

[75] Xie D., Yang Y., Cheng L. (2021). The impact of home quarantine and physical exercise on mental health during COVID-19. Chin. J. Clin. Psychol..

[76] Li F., Wang D. (2022). On the balance between public right to know and patients’ privacy right in COVID-19 prevention and control. Med. Jurisprud..

